# High efficacy of gefitinib in the treatment of EGFR mutation-positive advanced non-small cell lung adenocarcinoma: A case report

**DOI:** 10.3892/ol.2014.2269

**Published:** 2014-06-19

**Authors:** ZHONGCHAO WANG, JIANJUN CHU

**Affiliations:** 1 Department of Radiotherapy, Xinyi People’s Hospital, Xuzhou, Jiangsu 221400, P.R. China; 2 Department of Radiotherapy, The Fourth Affiliated Hospital of Soochow University, Wuxi, Jiangsu 214062, P.R. China

**Keywords:** gefitinib, epidermal growth factor receptor, tyrosine kinase inhibitors, non-small cell lung cancer

## Abstract

Epidermal growth factor receptor (EGFR) tyrosine kinase inhibitors (TKIs), such as gefitinib and erlotinib, are known to play a significant role in EGFR mutation-positive non-small cell lung cancer. When an EGFR mutation is found, gefitinib and erlotinib have been shown to have significant roles in the treatment of untreated advanced NSCLC. This study reports an EGFR mutation in NSCLC treated with gefitinib and is notable due to the patient’s marked improvement following a shorter than average duration of treatment with gefitinib. The present study reports the case of a 58-year-old male smoker with a dry cough. Computed tomography revealed a mass in the left inferior lobe of the lung. The patient was subsequently diagnosed with advanced lung adenocarcinoma, and an EGFR mutation (in-frame deletions of E746-A750 in exon 19) was found. The patient received multiple rounds of chemotherapy, followed by gefitinib maintenance therapy for 3 months. Later on, a grade 1 acne-like rash developed on the face and back that lasted throughout the treatment. Currently, the patient is stable, with no evidence of disease progression. The present study describes the disease and the treatment using gefitinib.

## Introduction

Non-small cell lung cancer (NSCLC) is a common cause of cancer-related mortality in China. Although several novel targeted anticancer agents are available, platinum-based chemotherapy remains the first-line therapy, achieving better progression free survival (PFS) rates than non-platinum-based regimens (1).

Epidermal growth factor receptor (EGFR) tyrosine kinase inhibitors (TKIs), such as gefitinib and erlotinib, have been shown to play a significant role in the treatment of untreated advanced NSCLC, particularly in NSCLC patients with EGFR mutations. Two phase III studies (NEJ002 and WJTOG3405) showed an improved PFS rate in NSCLC patients harboring sensitizing EGFR mutations (2,3). Therefore, gefitinib and erlotinib can be used as the first-line treatment of patients with advanced or metastatic NSCLC with activating EGFR mutations.

The present study describes the case of a patient with an EGFR mutation in NSCLC treated with gefitinib, achieving a marked efficacy. Patient provided written informed consent.

## Case report

A 58-year-old male, with no significant medical history developed a dry cough in November 2011. The patient had previously smoked 10 cigarettes per day for 30 years, but stopped smoking in January 2012. A chest computed tomography (CT) scan revealed a mass in the left inferior lobe, resulting in the patient being admitted to The Fourth Affiliated Hospital of Soochow University (Wuxi, Jiangsu, China). A brain CT scan showed no evidence of any distant metastasis. A bone emission CT (ECT) scan showed multiple bone metastases. Positron emission tomography-CT scan showed a large soft-tissue mass in the left inferior lobe of the lung, and multiple masses in the right lung, right adrenal glands and bones. Fiber bronchoscopy found cancer cells in the section analyzed, and histopathology revealed an adenocarcinoma. The patient was diagnosed with adenocarcinoma by a CT-guided percutaneous core needle biopsy. The clinical stage was stage IV. EGFR mutations were detected using the peptide nucleic acid-locked nucleic acid polymerase chain reaction clamp method. An EGFR mutation was found with deletions in E746-A750 of exon 19.

Due to a metastasis in the eleventh and twelfth thoracic vertebrae that caused spinal cord compression, the patient initially received 30 Gy radiation of 3 Gy per fraction. Following this, 1.6 g gemcitabine (GEM 1.0 g/m^2^) was administered on days one and eight, and 30 mg cisplatin (DDP) was administered on days one to four. Six cycles were administered every three weeks (February-July, 2012). No adverse events (AEs) were reported.

A chest CT scan carried out in August 2012 showed residual disease in the left inferior lobe of the lung (2 cm in diameter), and metastatic lesions of the right lung, the right adrenal glands and bone were stable. Chemotherapy was subsequently continued. The patient received four cycles of chemotherapy consisting of 1.6 g GEM on days one an eight, and 30 mg DDP on days one to four. The last chemotherapy treatment was on November 24, 2012. No AEs were reported. A CT scan of the thorax was assessed as stable. In May 2013, another ECT scan revealed one new lesion in the right femur, indicating progression of the disease. The patient received another 30 Gy radiation, 3 Gy/FX.

**Figure 1:**
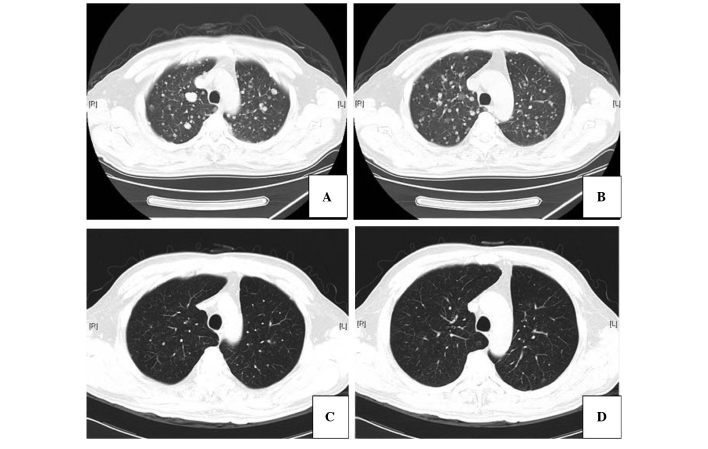
Axial chest computed tomography (CT) scans. (A and B) Scans prior to treatment with gefitinib. (C and D). Scans two months after treatment with gefitinib.

Another Chest CT scan showed widespread metastases in the right and left lung (Fig. 1). The patient was consequently administered 250 mg oral gefitinib once daily in June 2013. A grade 1 acne-like rash developed on the face and back, which was treated with 4.5 g piperacillin-tazobactam twice daily for 5 days. The rash lasted the clinical course of the treatment. Chest CT scans showed that the metastatic tumors were improved following gefitinib treatment (Fig. 1). To date, the disease remains stable and the patient continues to receive gefitinib orally.

## Discussion

The current study presents the case of an NSCLC patient with an EGFR mutation treated with gefitinib. For an unknown EGFR status, platinum-based chemotherapy remains in use as the first-line management of NSCLC (4,5). However, gefitinib is the first targeted agent to be approved for the treatment of EGFR mutation-positive lung adenocarcinoma, which has showed evident clinical efficacy, particularly among non-smokers, East Asian females and patients with adenocarcinoma (6,7). EGFR mutation analyses have demonstrated that patients with activating EGFR gene mutations obtained more benefit than those of non-EGFR gene mutations (8). A recent meta-analysis also showed that gefitinib yielded a statistically significant benefit in progression-free survival compared with gefitinib-free therapy (9).

Drug-related side-effects are significantly more common in patients receiving gefitinib therapy (9). EGFR-TKI therapy causes the development of a rash in numerous patients (10). In the patient of the present study, a grade 1 acne-like rash developed on the face and back following the start of gefitinib treatment. The rash persisted throughout the treatment period. Rash development has been associated with EGFR-TKI efficacy in NSCLC, and a meta-analysis previously showed that a skin rash was an independent predictive factor for progression and survival in EGFR TKI-treated NSCLC patients (10). Other adverse events, such as diarrhea, dry skin, pruritus and paronychia, have also been notable in the current literature. Therefore, the status of the patient should be considered prior to gefitinib treatment to ensure that the best therapy is being selected.

EGFR-TKI therapy may prolong life expectancy. Despite the fact that the treatment time is short, we believe that if used long-term, maintenance therapy would benefit those patients with EGFR mutations. Furthermore, a retrospective study analyzed 301 long-term NSCLC survivors to confirm which patient may benefit most from erlotinib treatment (11). The long-term gefitinib benefits were not only relative to good prognostic factors (e.g., good performance status, never-smoker status and the female gender), but also included negative factors. A study of 124 advanced NSCLC patients treated with chemotherapy [64 (51.6%) patients treated with gefitinib] as the initial therapy revealed that 8% of the patients survived for >5 years (12). In addition, another study reported that three refractory NSCLC patients survived for >3 years with gefitinib treatment (13). It was concluded that patients with EGFR mutations gained the greater benefit, particularly those who were older or had a poor performance status, from first-line EGFR-TKI therapy (12).

In conclusion, this study suggests that gefitinib therapy has a significant role in advanced lung adenocarcinoma. A limitation of the current study was the small sample size. Future studies with larger sample size are required to investigate EGFR mutations.
